# New Orleans School of Medicine

**Published:** 1860-08

**Authors:** 


### New Orleans School of Medicine.

The advertisement of the next course of Lectures in the New Orleans School of Medicine, will be found on the cover of this number of the Journal. Without intending to detract from the merits of any other Medical College in the Southern country, we shall not, yet, hesitate to say that in our judgment the student cannot do better than to avail himself of the advantages offered by this institution. New Orleans *must* and *should* become the great centre of medical teaching in the South, and, indeed, will, in all probability, in a very short time take precedence of New York and Philadelphia. In less than five years the *tide* of medical students from almost every Southern State, will be steadily set in the direction of New Orleans. There is no other point in the South which can furnish the advantages necessary for a *finishing* school, and while, doubtless, other colleges less favorably situated will be able to maintain a respectable position, New Orleans will and should be the great point of attraction, possessing, as she does, not only science and talent of the highest order, but offering Hospital and clinical advantages not furnished at any other point in the United States.

